# Comparative efficacy of quarterly vs. monthly triptorelin in Chinese girls with central precocious puberty: a 12-month observational study

**DOI:** 10.3389/fped.2026.1774350

**Published:** 2026-04-16

**Authors:** Hongyi Cai, Wenyong Wu, Xin Yuan, Ying Zhang, Xiaohong Yang, Xiaozhen Huang, Zhuanzhuan Ai, Dandan Li, Ruimin Chen

**Affiliations:** Department of Endocrinology, Genetics, and Metabolic Diseases, Fuzhou First General Hospital Affilliated with Fujian Medical University, Fuzhou Children’s Hospital, Fuzhou, China

**Keywords:** bone age, central precocious puberty, long-acting formulation, predicted adult height, triptorelin

## Abstract

**Background:**

This study aimed to compare the clinical efficacy of quarterly (15 mg/90 days) versus monthly (3.75 mg/28 days) triptorelin formulations with respect to bone maturation, predicted adult height (PAH), and hormonal suppression in Chinese girls with central precocious puberty (CPP).

**Methods:**

This retrospective cohort study included 70 girls diagnosed with CPP at Fuzhou First General Hospital from March 2023 to 2025. Participants were assigned to either a quarterly regimen (triptorelin 15 mg every 90 days, *n* = 32) or a monthly regimen (triptorelin 3.75 mg every 28 days, *n* = 38). The evaluated outcomes included bone age (BA; TW3 method), PAH, serum luteinizing hormone (LH) and follicle-stimulating hormone (FSH) concentrations, uterine and ovarian volumes, body mass index (BMI), and adverse events, assessed at baseline, 6 months, and 12 months.

**Results:**

After 12 months of treatment, both regimens achieved effective and comparable suppression of LH (quarterly: 0.30 ± 0.19 IU/L; monthly: 0.30 ± 0.26 IU/L; both *P* *<* 0.001 vs. baseline) and FSH (quarterly:1.22 ± 0.91 IU/L; monthly: 1.31 ± 0.65 IU/L; *P* *<* 0.05 vs. baseline). Improvements in PAH were also comparable between groups (quarterly: 3.95 ± 2.97 cm; monthly: 2.91 ± 2.70 cm; *P* *=* 0.790). No significant between-group differences were observed in BA maturation, growth velocity, or uterine and ovarian volumes (*P* *>* 0.05). BMI increased significantly in both groups (quarterly: from 16.96 ± 2.10 to 17.89 ± 2.53 kg/m^2^, *P* = 0.01; monthly: from 16.89 ± 2.06 to 17.50 ± 2.03 kg/m^2^, *P* = 0.008), with no significant differences between regimens. Three patients experiencing a “flare-up” had a larger mean baseline uterine volume (6.95 mL) compared with the remaining 67 patients (2.38 mL).

**Conclusion:**

Quarterly triptorelin demonstrated efficacy comparable to that of monthly administration in suppressing puberty and improving PAH in Chinese girls with CPP.

## Introduction

Central precocious puberty (CPP) results from premature activation of the hypothalamic-pituitary-gonadal axis (HPGA) (1), characterized by the onset of breast development before 8 years of age or menarche before 10 years of age (2). Epidemiological data indicate that the global incidence of CPP ranges from approximately 1:5,000–1:10,000 (3, 4), and has been increasing in recent years—potentially related to environmental, nutritional or genetic factors (5). Notably, CPP not only accelerates bone age (BA) maturation, resulting in compromised final adult height, but also may induce psychological and social adaptation challenges, such as anxiety and self-identity disorders, which may adversely impact the quality of life of affected children (6).

Gonadotropin-releasing hormone analogues (GnRHa) represent the mainstay of therapy for CPP, suppressing gonadotropin secretion to decelerate gonadal development and BA maturation (7). Triptorelin, a widely used GnRHa, is available in two intramuscular formulations in China: a monthly preparation (3.75 mg/month) and a quarterly preparation (15 mg/3 months). Notably, the quarterly formulation significantly improves treatment adherence due to reduced frequency of injections, rendering it particularly suitable for patients who are reluctant to undergo frequent injections (8). Although numerous international studies have validated the inhibitory effect of the quarterly formulation of triptorelin on the gonadal axis (9, 10), only one single-arm study in China has investigated its efficacy and safety in CPP treatment. To date, no studies have directly compared the efficacy of the quarterly and monthly triptorelin formulations in the Chinese population (9). Here, we compared the effects of these two regimens on BA inhibition, predicted adult height (PAH), and hormone levels, aiming to provide evidence for a safe, effective, and convenient quarterly treatment option.

## Materials and methods

### 1 Subjects

This study enrolled 70 girls diagnosed with CPP who received triptorelin for more than 12 months at the Department of Endocrinology, Genetics, and Metabolism, Fuzhou First General Hospital Affiliated to Fujian Medical University, Fuzhou Children’s Hospital of Fujian Province between March 2023 and March 2025. Inclusion criteria were as follows: (1) Girls with breast development (Tanner B2 stage) before 7.5 years of age or menarche before 10 years of age; baseline LH > 0.83 IU/L (chemiluminescence) (11), or peak LH ≥ 5.0 U/L with LH/FSH ratio ≥ 0.6 during a standard GnRH stimulation test (11, 12); height velocity >6 cm/year and BA at least 12 months older than chronological age; ultrasound findings showing uterine length > 3.7 cm, ovarian volume ≥1 mL, and multiple follicles ≥4 mm in diameter (12); and no organic lesions identified by brain magnetic resonance imaging (MRI). (2) No prior GnRHa treatment. (3) Duration of GnRHa treatment ≥12 months. Exclusion criteria were as follows: (1) Comorbidity with chronic diseases or other endocrine abnormalities. (2) Loss to follow-up or incomplete case data. (3) Administration of growth hormone or other drugs that affect the growth process at the initiation or during the course of treatment. (4) Any unplanned modification of the dosing regimen or poor compliance during the course of treatment.

**Figure 1:**
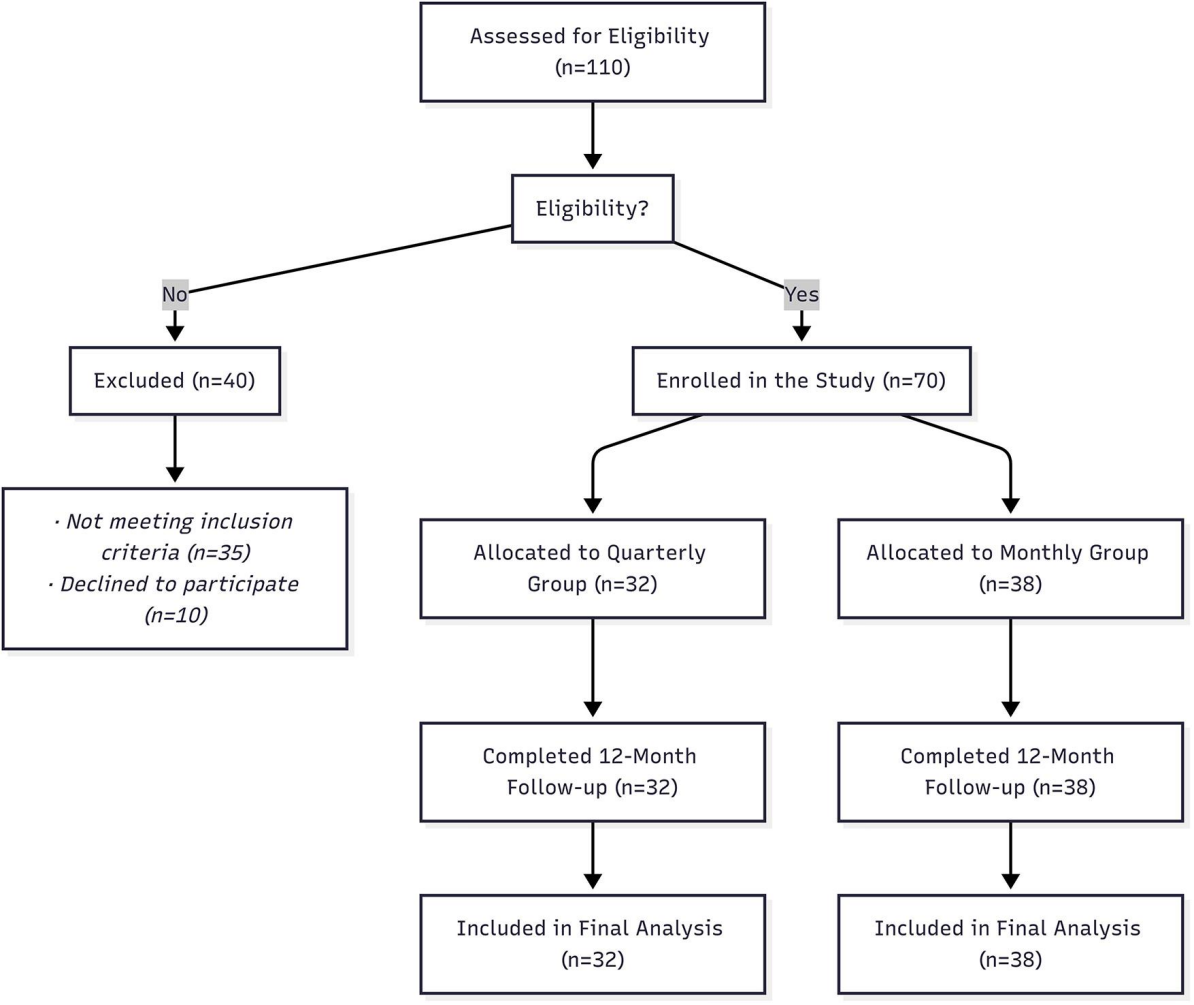
Patient disposition.

This study was a real-world, retrospective cohort study designed to evaluate the effectiveness of two triptorelin formulations as administered in routine clinical practice. Patients were not randomly allocated to the quarterly or monthly treatment groups; instead, assignment was based on shared decision-making with families. The choice between formulations depended on parental preferences, upfront costs versus reduced travel frequency, and geographic distance to our centre. This non-randomized allocation reflects the real-world clinical decision-making. To ensure transparency and mitigate potential selection bias, we present a detailed comparison of all key baseline characteristics and provide a participant flow diagram (Figure 1) outlining the screening and enrollment process.

### 2 Methods

Girls with CPP were divided into two groups according to the treatment regimen: the quarterly group received intramuscular triptorelin at a dose of 15 mg every 90 days, and the monthly group received intramuscular triptorelin at a dose of 3.75 mg every 28 days. All enrolled patients had a body weight strictly >20 kg. Drug dosages for both formulations were administered strictly according to clinical guidelines and manufacturer instructions (13), without dose adjustments based on individual weight changes. All intramuscular injections were administered by experienced pediatric nurses in the outpatient clinic of our hospital to ensure proper administration and strict adherence.

Follow-up visits included regular monitoring of height and weight, as well as measurement of serum LH and FSH levels every 3 months. Utero-ovarian ultrasonography and left wrist radiographs were performed at 6-month intervals, with BA assessed using the Tanner-Whitehouse 3 (TW3) method every 6 months (14). The TW3 method was selected for its high reproducibility and common use in international studies, facilitating direct comparisons. Predicted adult height (PAH) was calculated utilizing the TW3 method’s multiple regression equations. The following linear regression model was applied: PAH = Current Height + *β*_1_·(Chronological Age) + *β*_2_·(TW3-RUS Score) + C. The TW3-RUS score represents the sum of maturity scores of the radius, ulna, and short bones. The regression coefficients (*β*_1_ and *β*_2_) and constant (C) are explicitly age- and sex-specific, derived directly from the standard TW3 reference manual (15).

At each visit, detailed medical history and physical examination findings were recorded. Variables assessed at baseline and at 6 and 12 months included chronological age (CA), parental heights, height, weight, BMI, BA/CA ratio, Tanner stage for breast and pubic hair development, basal and peak LH and FSH levels, BA, PAH, uterine and ovarian volumes, and drug-related adverse events. Adequate pubertal suppression was defined as a peak LH <2.5 IU/L. This threshold is assay-specific. Older guidelines using radioimmunoassays cited thresholds of <3.0 or <4.0 IU/L, but the cutoff of <2.5 IU/L is optimal and widely adopted in recent clinical trials employing sensitive chemiluminescence assays (8, 16). A “flare-up” was clinically defined as transient vaginal bleeding or spotting within the first 4 weeks after treatment initiation. It results from the initial physiological stimulation of the HPGA prior to receptor downregulation.

Height and weight were measured by trained nurses using the same calibrated stadiometer and scale, with measurements conducted at approximately the same time of day whenever possible. BMI was calculated as weight (kg) divided by height squared (m^2^). Target height (TH) for girls was estimated using the formula: (father’s height + mother’s height − 13)/2. Breast and pubic hair development were assessed using Tanner staging. All girls except those with basal LH >0.83 IU/L underwent a GnRH stimulation test with intravenous gonadorelin at baseline and 3 months post-treatment. Serum LH and FSH levels were measured by chemiluminescence. Uterine and ovarian volumes were calculated using the formula *π*/6 × length × width × thickness. BA was assessed serially by an independent pediatric endocrinologist who was blinded to the patients’ treatment allocations, using the TW3 method. All ultrasounds were performed by two experienced pediatric sonographers following a standardized protocol.

### 3 Statistical analyses

Statistical analyses were performed using SPSS version 23.0. Before formal analysis, all variables underwent rigorous testing for normality and homogeneity of variance. In this study, all continuous variables met these statistical assumptions; therefore, comparisons between the two independent groups were conducted exclusively using parametric independent samples t-tests. Results are presented as mean ± standard deviation (SD). A two-sided significance threshold of *α* = 0.05 was applied, and *P*-values <0.05 were considered statistically significant.

## Results

Of the enrolled cohort, 32 patients were assigned to the quarterly group and 38 to the monthly group. At baseline, there were no significant differences between the two groups in CA, height, weight, BMI, serum LH and FSH levels, BA, BA/CA ratio, or PAH (all *P* *>* 0.05) (Table 1).

**Table 1:** Baseline characteristics of monthly and quarterly triptorelin groups.

Variable	Quarterly (*n* = 32)	Monthly (*n* = 38)	*P*
CA (year)	8.49 ± 0.95	7.67 ± 0.86	0.299
Height (cm)	133.99 ± 5.61	129.98 ± 6.98	0.234
Weight (kg)	30.64 ± 5.53	28.72 ± 5.38	0.923
BMI (kg/m^2^)	16.96 ± 2.10	16.89 ± 2.06	0.907
LH (IU/L)	1.53 ± 2.55	1.27 ± 1.82	0.381
FSH (IU/L)	3.31 ± 1.76	3.45 ± 1.88	0.723
BA (year)	11.02 ± 0.86	10.56 ± 1.09	0.304
BA/CA	1.31 ± 0.10	1.38 ± 0.11	0.405
TH (cm)	158.35 ± 3.37	157.12 ± 4.70	0.055
PAH (cm)	148.68 ± 3.84	147.66 ± 2.90	0.255
Uterine volume (ml)	3.20 ± 4.38	2.72 ± 2.57	0.220
Left ovarian volume (ml)	2.27 ± 1.64	2.46 ± 1.71	0.287
Right ovarian volume (ml)	2.43 ± 1.86	2.37 ± 1.40	0.692

Quarterly, quarterly group; Monthly, monthly group; CA, chronological age; BMI, body mass index; LH, luteinizing hormone; FSH, follicle stimulating hormone; BA, bone age; PAH, predicted adult height.

At 3 months, there were no significant differences between the quarterly and monthly groups in basal LH (0.30 ± 0.17 vs. 0.31 ± 0.12 IU/L, *P* = 0.200) or basal FSH (0.88 ± 0.86 vs. 0.77 ± 0.36 IU/L, *P* = 0.092) levels. Both groups exhibited significant suppression of LH and FSH, with peak LH levels remaining below 2.5 IU/L during the gonadorelin stimulation test (0.56 ± 0.35 vs. 0.44 ± 0.26 IU/L, *P* *=* 0.189). At 6 and 12 months, basal plasma LH and FSH concentrations were significantly reduced compared with baseline in both groups (*P* *<* 0.001). Additionally, there were no significant differences in LH and FSH levels between the two groups at 6 and 12 months of treatment (Table 2). Serum estradiol (E2) levels in all patients decreased to prepubertal levels (≤20 pg/mL) at 3, 6, and 12 months.

**Table 2:** Data in CPP girls during and after triptorelin treatment.

	At 6 months			At 12 months		
Variable	Quarterly (*n* = 32)	Monthly (*n* = 38)	*P*	Quarterly (*n* = 32)	Monthly (*n* = 38)	*P*
Height (cm)	137.17 ± 5.47	133.71 ± 7.08	0.225	139.90 ± 5.38	136.07 ± 6.39	0.422
Weight (kg)	32.66 ± 6.00	30.84 ± 5.73	0.751	35.27 ± 6.94	32.55 ± 5.50	0.145
BMI (kg/m^2^)	17.25 ± 2.18	17.14 ± 2.05	0.552	17.89 ± 2.53	17.50 ± 2.03	0.271
Basal LH (IU/L)	0.29 ± 0.19	0.28 ± 0.15	0.206	0.30 ± 0.19	0.30 ± 0.26	0.601
Basal FSH (IU/L)	0.95 ± 0.87	0.82 ± 0.40	0.099	1.22 ± 0.91	1.31 ± 0.65	0.303
BA (year)	11.40 ± 0.81	10.92 ± 1.11	0.124	11.73 ± 0.80	11.23 ± 1.07	0.166
BA/CA (year)	1.26 ± 0.09	1.33 ± 0.11	0.277	1.24 ± 0.08	1.29 ± 0.10	0.177
Growth velocity (cm/years)	6.36 ± 1.52	7.45 ± 2.10	0.170	5.71 ± 1.54	6.18 ± 1.84	0.863
Uterine volume (ml)	1.52 ± 0.58	1.56 ± 0.98	0.220	1.72 ± 1.08	1.78 ± 1.03	0.454
Left ovarian volume (ml)	1.47 ± 0.66	1.57 ± 0.75	0.074	2.03 ± 1.11	1.69 ± 0.94	0.570
Right ovarian volume (ml)	1.51 ± 0.72	1.60 ± 0.84	0.307	1.95 ± 1.06	1.86 ± 1.17	0.401

Quarterly, quarterly group; Monthly, monthly group; LH, luteinizing hormone; FSH, follicle stimulating hormone; BA, bone age; CA, chronological age; BMI, Body Mass Index; PAH, predicted adult height.

During the treatment period, growth velocity in the quarterly group was commensurate with that observed in the monthly group. Compared with baseline, PAH improved significantly in both groups at 6 and 12 months; after 12 months, the mean PAH was 152.33 ± 4.09 cm in the quarterly group and 150.56 ± 3.93 cm in the monthly group. At any time points, the difference in PAH between the two groups was not significant (Table 3). After 12 months of treatment, BMI increased in both groups, from 16.96 ± 2.10 kg/m^2^ and 16.89 ± 2.06 kg/m^2^ before treatment to 17.89 ± 2.53 kg/m^2^ and 17.50 ± 2.03 kg/m^2^, respectively (*P* = 0.01 and 0.008). In addition, there was no overt progression in breast and pubic hair Tanner staging in either group.

**Table 3:** PAH before and after triptorelin treatment.

Variable	Quarterly (*n* = 32)	Monthly (*n* = 38)	*P*
PAH (cm)			
Baseline	148.38 ± 3.31	147.66 ± 2.90	0.615
6 months post treatment	150.35 ± 3.49	149.61 ± 3.39	0.874
12 months post treatment	152.33 ± 4.09	150.56 ± 3.93	0.990
ΔPAH (cm)			
6 months post treatment	1.97 ± 1.71	1.95 ± 1.72	0.870
12 months post treatment	3.95 ± 2.97	2.91 ± 2.70	0.790

Quarterly, quarterly group; Monthly, monthly group; PAH, predicted adult height.

**Figure 2:**
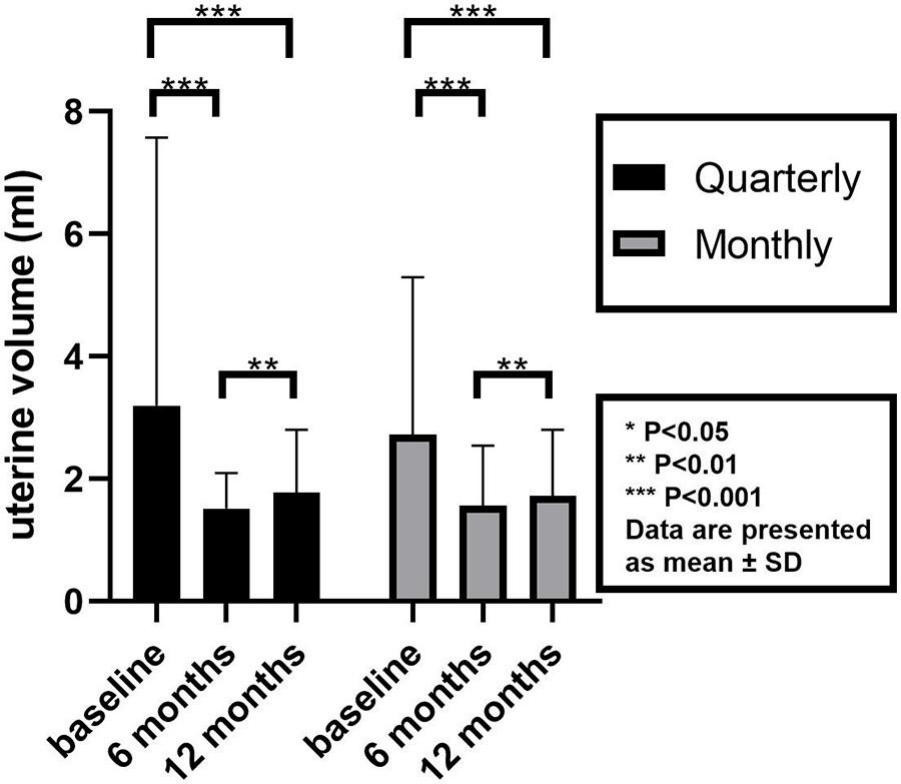
Uterine volume before and after triptorelin treatment.

After 6 months of treatment, BA was 11.40 ± 0.81 years in the quarterly group and 10.92 ± 1.11 years in the monthly group; at 12 months, BA increased to 11.73 ± 0.80 years and 11.23 ± 1.07 years, respectively. No statistically significant differences in BA were observed between the two groups at any time point (Table 2). Uterine volume decreased significantly from baseline at both 6 and 12 months. However, a modest but statistically significant increase in uterine volume was noted between the 6- to 12-month measurements (Figure 2).

A total of 8 adverse drug reactions (ADRs, 11.4%) were documented (Table 4). Additionally, 4 cases of upper respiratory tract infections were observed, which were deemed unrelated to treatment. Three patients who developed a “flare-up” following treatment had baseline uterine volumes of 4.32, 5.86, and 10.68 mL (mean 6.95 mL), while the remaining 67 girls had a mean baseline volume of 2.38 mL. Importantly, initial “flare-ups” did not correlate with hormonal escapes, as all affected patients achieved and maintained complete LH suppression (<2.5 IU/L) by the 3-month follow-up and thereafter. All ADRs occurred during the initial treatment phase, were non-serious, and did not necessitate dose reduction or trial withdrawal.

**Table 4:** ADRs in CPP girls during triptorelin treatment.

Variable	Participants	Time from first injection	Duration (day)	CTCAE Grade
Quarterly				
“flare-up”	2/32 (6.3%)	15th/20th day	3/5	CTCAE Grade 1: light bleeding, no sanitary napkins required or only panty liners needed.
myalgia	2/32 (6.3%)	1st/7th day	3 1/2	CTCAE Grade 1: mild pain without functional impairment.
Monthly				
“flare-up”	1/38 (2.6%)	3rd day	1	CTCAE Grade 1: light bleeding, no sanitary napkins required or only panty liners needed.
myalgia	1/38 (2.6%)	5th day	3	CTCAE Grade 1: mild pain without functional impairment.
asthenia	1/38 (2.6%)	4th day	2	CTCAE Grade 1: fatigue relieved by rest, no interference with daily activities.
dizziness	1/38 (2.6%)	1st	1	CTCAE Grade 2: transient vertigo with brief positional aggravation, requiring non-pharmacological intervention but no treatment discontinuation.

ADRs, adverse drug reactions; Quarterly, quarterly group; Monthly, monthly group; CTCAE, common terminology criteria for adverse events.

## Discussion

This study provides real-world comparative data on the 12-month efficacy of quarterly and monthly triptorelin formulations in Chinese girls with CPP. After one year of treatment, we found no significant differences between the two groups in height, BA, uterine ultrasound parameters, or HPGA hormone levels, suggesting comparable efficacy. These findings are consistent with previous reports. Bertelloni et al. and Lee et al. (17, 18) enrolled 25 (13 quarterly, 12 monthly) and 69 (29 quarterly, 40 monthly) girls with CPP, respectively, who were treated with triptorelin. Comparisons between the two treatment groups—including height, weight, BMI, BA, BA/CA ratio, PAH, PAH improvement, and growth velocity—revealed no significant differences in efficacy between the two formulations, supporting the results observed in our cohort.

Both regimens were effective in suppressing LH and FSH secretion, as well as breast development. Park et al. (10) reported that at 6 months, LH and FSH levels were similarly reduced in both treatment groups, consistent with our results. No significant differences were observed between the two groups, indicating that pubertal suppression was achieved by 3 months for both formulations (19, 20). Furthermore, breast Tanner staging remained stable throughout 6 and 12 months of treatment, further validating the inhibitory effect of triptorelin on pubertal progression.

In our cohort, both quarterly and monthly groups showed a statistically significant increase in uterine volume between 6 and 12 months of treatment. B. Liebezeit et al. (21) retrospectively evaluated 27 girls with CPP who had received triptorelin or leuprolide treatment, reporting that 44% of participants experienced a further increase in uterine volume between 6 and 12 months. In our cohort, this slight enlargement did not correlate with LH escapes, as all patients maintained LH levels <2.5 IU/L. This finding suggests the enlargement reflects normal physiological processes rather than central therapy escape. Importantly, all studies consistently demonstrated effective suppression of uterine and ovarian volumes one year after treatment (22, 23). Hence, uterine and ovarian volume alone should not be considered definitive indicators of therapeutic efficacy.

The efficacy of quarterly and monthly triptorelin on growth was evaluated by various indicators such as BA, PAH, and growth velocity. Both formulations were effective in suppressing BA and improving height gain, with no significant differences observed between the two groups. In a study of 150 girls with CPP in Korea (10), the quarterly formulation effectively inhibited BA maturation and improved PAH. Similarly, Bertelloni et al. (17) reported no significant differences in height, BA, and PAH between girls treated with monthly or quarterly triptorelin, findings that are consistent with our results (24–26). Furthermore, Bertelloni et al. (17) showed that there were no significant differences in final adult height between the two treatment regimens. Given that the 3-month triptorelin depot was only recently introduced in China, none of the girls in our cohort have reached their FAH. We acknowledge that using PAH as a surrogate marker constitutes a limitation of this study. Longitudinal follow-up is necessary to confirm the FAH outcomes. Regarding growth-related parameters, BMI increased significantly during triptorelin treatment in our cohort, which is in line with most previous studies (9, 27).

In this study, patients who experienced vaginal bleeding after treatment had baseline uterine volumes higher than the group average. After 3 months of treatment, uterine volumes decreased significantly and remained suppressed at 6 and 12 months after treatment. However, ultrasound evaluations were not performed promptly at the time of the “flare-up” bleeding events. Although De Sanctis et al. (28) reported self-limiting vaginal bleeding events associated with GnRHa therapy, their review did not analyze the correlation with uterine volume. Whether uterine developmental status is associated with the risk of GnRHa-related “flare-up” bleeding remains to be validated in large-scale studies. Regarding adverse events, a single-arm study by Luo et al. (9) reported that 31.3% of CPP patients experienced upper respiratory tract infection-related adverse events, with 6.3% deemed drug-related. Such discrepancies may be attributable to differences in environmental exposure, seasonal follow-up timing, or monitoring sensitivity. In the present cohort, only mild systemic adverse reactions were recorded, with no sterile abscesses occurring or allergic reactions. The severity of reactions was substantially lower than the 0.9% rate of severe ADRs reported by Lee et al. (29), which included 0.6% sterile abscesses and 0.16% anaphylactic shock. While ADR profiles varied slightly between formulation groups, De Sanctis et al. (28) highlighted that individual susceptibility, rather than the dosing interval, is the primary determinant of adverse events.

Notably, the triptorelin 15 mg/90 days formulation used in our study is pharmacologically equivalent to the internationally cited 11.25 mg/90 days formulation. The difference in labeled strengths merely reflects regional regulatory labeling practices based on total salt weight versus active peptide base. Previous studies have confirmed their identical safety and efficacy profiles, ensuring our outcomes are directly comparable with global findings (30, 31). Methodologically, our diagnostic age threshold of 7.5 years aligns with the 2022 Chinese consensus and satisfies classic international criteria, ensuring that our cohort is highly representative and globally comparable. Similarly, we deliberately utilized the TW3 method for bone age assessment, rather than regional standards like CHN05, to reliably track longitudinal trends and facilitate direct comparisons with international cohorts.

This study has several limitations that should be acknowledged. First, its retrospective and non-randomised design, together with the relatively modest sample size, may limit statistical power, particularly for detecting subtle growth changes or less frequent adverse events. Furthermore, the 12-month follow-up period may not be sufficient to fully characterise long-term efficacy; therefore, extended longitudinal follow-up until FAH is warranted to provide more robust evidence.

## Conclusions

In our study, quarterly triptorelin demonstrates comparable efficacy to the monthly formulation in suppressing pubertal progression and improving PAH in Chinese girls with CPP. Given its efficacy and the reduced frequency of injections, the quarterly triptorelin represents a practical and convenient treatment option for CPP.

## Data Availability

The raw data supporting the conclusions of this article will be made available by the authors, without undue reservation.
